# Serum Sialic Acid Level Is Significantly Associated with Nonalcoholic Fatty Liver Disease in a Nonobese Chinese Population: A Cross-Sectional Study

**DOI:** 10.1155/2016/5921589

**Published:** 2016-02-10

**Authors:** Zhenya Lu, Han Ma, Chengfu Xu, Zhou Shao, Chao Cen, Youming Li

**Affiliations:** ^1^Department of Internal Medicine, The First Affiliated Hospital, College of Medicine, Zhejiang University, Hangzhou 310003, China; ^2^Department of Gastroenterology, The First Affiliated Hospital, College of Medicine, Zhejiang University, Hangzhou 310003, China; ^3^Division of Hepatobiliary and Pancreatic Surgery, Department of Surgery, The First Affiliated Hospital, College of Medicine, Zhejiang University, Hangzhou 310003, China

## Abstract

*Background/Aim*. To investigate the association between serum sialic acid (SA) levels and nonalcoholic fatty liver disease (NAFLD) in a nonobese Chinese population.* Methods*. A cross-sectional study was performed among the 5916 adults who took their annual health examinations at International Health Care Center, The First Affiliated Hospital, College of Medicine, Zhejiang University, from December 2013 to November 2014.* Results*. A total of 693 (11.71%) subjects fulfilled the diagnostic criteria of NAFLD, and NAFLD patients had significantly higher serum SA levels than controls (*P* < 0.001). The prevalence of NAFLD was positively associated with serum SA levels (*P* for trend <0.001). Serum sialic acid levels are significantly associated with features of metabolic syndrome (*Ps* < 0.01). Multivariate logistic regression analysis showed that serum SA level was significantly associated with risk for NAFLD (odds ratio: 1.018, 95%; confidence interval: 1.007–1.030; *P* = 0.002).* Conclusions*. Our results suggest for the first time that NAFLD patients had higher serum SA level than controls, and increased serum SA level is significantly associated with risk for NAFLD in a large nonobese Chinese population.

## 1. Introduction

Nonalcoholic fatty liver disease (NAFLD) is one of the most common chronic liver diseases worldwide [1]. NAFLD affects about 15–20% adults in China and more than 30% in developed countries [2]. The spectrum of NAFLD includes simple steatosis, steatohepatitis, fibrosis, and hepatic carcinoma. Simple steatosis is a benign condition with slow progression over years, while nonalcoholic steatohepatitis (NASH) may progress into end stage liver disease [3, 4]. NASH has been recently recognized to be the second leading reason for liver transplantation in the United States [5].

The development of NAFLD is closely associated with obesity and related metabolic disorders [6, 7]. Obesity is a major risk factor for NAFLD [6]. However, less attention has been paid to NAFLD in nonobese subjects. Actually, NAFLD is not scarce in nonobese adults. A recent cross-sectional study by our group revealed that the prevalence of NAFLD was 7.27% in nonobese Chinese population. The following prospective study showed that 8.88% subjects developed NAFLD in a 5-year duration [8]. Another study by Kim et al. reported that the prevalence of NAFLD was as high as 23.4% among 768 nonobese, nondiabetic individuals older than 30 years in Korea [9]. Thus, in order to better manage and prevent NAFLD, the factors that are related to presence and development of NAFLD in nonobese subjects should be taken into account.

Sialic acids (SA) are a diverse family of sugar units with a nine-carbon backbone that typically occupies the terminal position on the carbohydrate chains of glycoproteins and glycolipids [10, 11]. SA are mainly found at the outmost end of glycan chains of all cell type [11]. Total SA is a sum of protein-bound, lipid-bound, and free SA [12]. Given the location and ubiquitous distribution, SA can mediate or modulate various physiological and pathological processes, including obesity and metabolic syndrome. An increase in serum total SA is observed in obese subjects compared with nonobese controls [13]. Besides, SA correlates to metabolic syndrome components and predicts the risk of incident metabolic syndrome, independently of body mass index [14, 15].

Even though the close association between SA and obesity has been well established, the role of serum SA level in NAFLD, especially in nonobese individuals, has not been clarified so far. This study aimed to investigate the association of serum SA levels with NAFLD in a nonobese Chinese population.

## 2. Materials and Methods

### 2.1. Study Population

This study was performed among the adults who took their health checks at International Health Care Center, The First Affiliated Hospital, College of Medicine, Zhejiang University, from December 2013 to November 2014. Participants who had full records of personal healthy history, anthropometric and biochemical data, and results of hepatic ultrasonography were initially enrolled. Those who met the following criteria were excluded: (1) alcohol consumption greater than 140 g/week for males and 70 g/week for females; (2) a history of viral hepatitis, autoimmune hepatitis, or other forms of chronic liver disease; (3) body mass index (BMI) higher than 23 kg/m^2^. A total of 5916 participants (2310 men and 3606 women) with a mean (standard deviation) age of 43.3 (11.2) years were included in the final analysis.

Due to the observational nature of the study, written informed consent was not required for this study. All participants were verbally informed about the study and agreed to participate in the study. The participant information was anonymized at collection and anonymized prior to analysis. This study was approved by the Ethics Committee of The First Affiliated Hospital, College of Medicine, Zhejiang University.

### 2.2. Clinical Examinations

Clinical examinations were performed using standard procedures as we described previously [16, 17]. Height and body weight without shoes and with light clothing were measured. BMI was calculated as the weight (kg) divided by the square of the height (meters). Waist circumference was measured with a nonstretchable standard tape at the level at the narrowest point between the iliac crest and the rib cage. Blood pressure was recorded using an automated sphygmomanometer.

After an overnight fast, blood samples were obtained from each participant and the samples were used for biochemical analysis without freezing. The biochemical analysis was carried out by a Hitachi 7600 autoanalyzer (Hitachi, Tokyo, Japan) using standard methods. Total serum SA levels were measured by an enzymatic method using a commercial kit provided by Wenzhou Dongou Bioengineering Inc., China. The interassay coefficient of variation was less than 5%, and the intra-assay coefficient of variation was less than 10%.

### 2.3. Diagnosis of NAFLD

NAFLD was diagnosed based on the results of hepatic ultrasound examination following exclusion of alcohol consumption, viral, or autoimmune liver disease. The criteria for ultrasonic diagnosis of fatty liver were based on those proposed by the Chinese Liver Disease Association [18]. Fatty liver was diagnosed if ultrasonography showed diffuse enhancement of near field echo in the hepatic region and gradual attenuation of the far field echo, combined with any of the following manifestations: (i) unclear display of intrahepatic lacuna structure; (ii) mild to moderate hepatomegaly with a round and blunt border; (iii) color Doppler ultrasonography that shows a reduction of the blood flow signal in the liver or it is even hard to display, but the distribution of blood flow is normal [18]. Hepatic ultrasound examinations were carried out by trained ultrasonographists, who were blinded to the study design and clinical data, using an ACUSON Sequoia 512 ultrasound machine with a 3.5 MHz probe (Siemens, Mountain View, CA).

### 2.4. Statistics Analysis

Statistical analyses were carried out using SPSS 13.0 software for Windows (SPSS Inc., Chicago, IL). Continuous variables were expressed as mean and standard deviation or median and interquartile range and compared through the use of Student's *t*-test or Mann-Whitney *U* test. Categorical variables were compared using chi-square test. Pearson's or Spearman's analysis was applied to determine correlations between serum SA levels and other metabolic parameters. A multiple stepwise regression analysis (backward: Wald; cutoff for entry: 0.05, for removal: 0.10) was applied to assess the risk factors for NAFLD. *P* < 0.05 (2-tailed) was considered to be statistically significant.

## 3. Results

### 3.1. Clinical Characteristics of the Study Population

Of 5916 participants (2310 men and 3606 women, with mean age of 45.0 ± 12.1 and 42.2 ± 10.5, resp.) who met the inclusion criteria in this study, 693 (11.71%) fulfilled diagnostic criteria of NAFLD. The clinical characteristics of participants according to presence of NAFLD were listed in Table 1. Participants with NAFLD were older and male predominant and had higher BMI, waist circumference, and systolic and diastolic blood pressure than those without NAFLD (Table 1). The NAFLD patients also had higher serum levels of alanine aminotransferase, *γ*-glutamyltransferase, triglyceride, total cholesterol, LDL cholesterol, fasting plasma glucose, and serum uric acid, while having lower serum HDL cholesterol levels than NAFLD-free participants (all with *P* < 0.001; Table 1). These results suggest that, despite all participants being nonobese in this study, participants with NAFLD are associated with more unfavorable metabolic profiles than those without NAFLD.

### 3.2. Serum SA Levels Are Positively Associated with Prevalence of NAFLD

As illustrated in Table 1, serum SA levels are significantly elevated in NAFLD patients, compared with NAFLD-free controls (61.1 ± 7.3 versus 58.2 ± 6.9 mg/dL; *P* < 0.001; Table 1). This result suggests a potential association of serum SA with NAFLD. To investigate the association of serum SA with prevalence of NAFLD, we divided all the participants into quartiles according to their serum SA levels: <54.1, 54.1–58.7, 58.8–63.6, and ≥63.7 mg/dL. We observed a linear correlation between serum SA quartiles and prevalence of NAFLD. The prevalence was 7.27% among the participants with serum SA in the first quartile and increased to 8.96%, 11.85%, and 18.70%, in quartiles 2, 3, and 4, respectively (*P* for trend < 0.001; Table 2). These results indicate that participants with higher serum SA levels are more likely to have NAFLD.

### 3.3. Serum SA Levels Are Correlated with Features of Metabolic Syndrome

NAFLD is closely associated with metabolic syndrome. We performed correlation analysis to determine the correlations between serum SA levels and features of metabolic syndrome. We found that serum SA levels were positively correlated with waist circumference (*r* = 0.196, *P* < 0.001), systolic blood pressure (*r* = 0.175, *P* < 0.001), diastolic blood pressure (*r* = 0.168, *P* < 0.001), triglycerides (*r* = 0.212, *P* < 0.001), and fasting blood glucose (*r* = 0.159, *P* < 0.018), while negatively correlated with HDL-C (*r* = −0.077, *P* < 0.001) (Table 3). These results not only suggest a significant association between serum SA and metabolic syndrome, but also indirectly indicate a close relation between serum SA and NAFLD, which is a hepatic manifestation of metabolic syndrome.

### 3.4. Elevated Serum SA Levels Significantly Increase Risk of NAFLD

We further performed multivariable logistic regression analysis to investigate factors associated with NAFLD. We set NAFLD as the dependent variable and variables that are significantly different between NAFLD and controls to be predicator variables. The predicator variables include age, gender, BMI, waist circumference, systolic and diastolic blood pressure, alanine aminotransferase, *γ*-glutamyltransferase, triglyceride, total cholesterol, LDL and HDL cholesterol, fasting plasma glucose, serum uric acid, and SA. Except gender, the other 14 predicator variables were continuous. We found that nine variables remained in the final equation (Table 4), indicating that those variables are closely associated with risk for NAFLD. A noticeable finding was that serum SA was significantly associated with risk for NAFLD (OR = 1.018; 95% CI: 1.007–1.030; *P* = 0.002). This finding further supports a significant association between serum SA and NAFLD.

## 4. Discussion

In this study, we found that serum SA levels are significantly associated with NAFLD in a large nonobese Chinese population. First, serum SA levels are significantly elevated in NAFLD patients, and the levels are positively associated with prevalence of NAFLD. Second, serum SA levels are positively associated with features of metabolic syndrome, which indirectly suggests a close relation between serum SA and NAFLD. Third, regression analysis showed that elevated serum SA levels significantly increase risk of NAFLD.

The relationship between serum SA and NAFLD can be explained by the following reasons. First, insulin resistance is a central component of NAFLD [19]. Several studies have demonstrated that insulin resistance is associated with increased serum total SA level [20, 21]. Insulin resistance might elevate SA levels either by abnormal sialylation of glycoproteins and glycolipids or by inducing acute phase response [22, 23]. Based on this point, elevated serum SA levels in NAFLD in this study can be stated as the increase of insulin resistance in NAFLD patients.

The second possible mechanism by which serum SA linked with NAFLD is through oxidative stress. Oxidative stress has long been recognized to be central to the liver damage and disease progression in NAFLD [24]. Targets for the reactive oxygen species might be nonreducing terminal sialic acid residues, proteins, lipids, and DNA [25]. In response to oxidative stress, cell membranes break down. SA are mainly located at the outmost end of glycan chains of all cell types [11]; the released SA would enter the blood stream with glycolipids and thereby elevate serum SA levels. Given this phenomenon, the elevated serum SA levels have been considered a reflection of altered structural integrity of glycolipids in cell membranes [26]. However, at the same time, the released SA would in return attenuate oxidative stress, acting as a reactive oxygen scavenger [27].

The third explanation is inflammation. A growing body of evidence supports a central role of inflammation in the pathogenesis of the NAFLD [24]. SA is component of some acute phase protein terminal part, which together explain 70% of SA plasmatic concentration [28]. The sialylation patterns of glycoproteins and glycolipids are highly variable and depend on the liver status [29]. Inflammation would increase the sialylated acute phase protein, which is produced by the liver in response to proinflammatory cytokines [30]. SA was found to be increased in many inflammatory conditions as well, such as obesity and cardiovascular diseases.

The link between serum SA and NAFLD may provide a potential explanation for why NAFLD can be a risk for cardiovascular diseases. Independent association between NAFLD and cardiovascular disease was emphasized in several epidemiological studies [31, 32]. However, the physiological mechanism for the link between NAFLD and cardiovascular disease remains unsolved. Since SA is indicated to be a marker for the risk of type 2 diabetes and cardiovascular mortality, independently of BMI [21, 28], the elevated serum SA reported in nonobese NAFLD patients in the present study may partially interpret why NAFLD increases the risk of cardiovascular disease. Special attention should be paid to the strategies that aim at monitoring and decreasing serum SA level to reduce risk of cardiovascular disease in NAFLD patients.

There are limitations in our study. The diagnosis of NAFLD was based on ultrasonographic method, which is not sensitive enough to detect mild steatosis. Although ultrasonography is reasonably accurate, noninvasive, and widely used for population-based studies [33], the cause-consequence relationship between serum SA and NAFLD could not be resolved by this cross-sectional study. Besides, our initial study design did not allow for the examination of insulin resistance and markers of oxidative damage, such as malondialdehyde (MDA), although insulin resistance and oxidative stress situation may closely be associated with NAFLD in nonobese subjects [24, 34]. Moreover, our results should be interpreted with caution. The values of most clinical indicators are within normal range and slightly, but statistically, significantly different between NAFLD patients and controls. The OR value is slightly higher than 1, indicating that SA may not be a key factor for NAFLD. Further studies are needed to clarify these issues.

In conclusion, we provide evidences for the first time that serum SA levels are significantly associated with NAFLD in a large nonobese Chinese population. Further studies are needed to explore the detailed relationship and the possible mechanisms between SA and NAFLD.

## Figures and Tables

**Table 1:** Characteristics of study participants with or without NAFLD.

Variables	With NAFLD (*n* = 693)	Without NAFLD (*n* = 5223)	*t* value	*P* value
Age (year)	48.0 (10.9)	42.7 (11.1)	11.856	<0.001
Gender (male/female, *n*)	404/289	1906/3317	122.222^a^	<0.001
Body mass index (kg/m^2^)	21.57 (1.45)	20.73 (1.56)	13.501	<0.001
Waist circumference (cm)	80.3 (6.3)	75.1 (6.6)	19.602	<0.001
Systolic blood pressure (mmHg)	124.9 (16.7)	118.1 (15.8)	10.496	<0.001
Diastolic blood pressure (mmHg)	76.3 (11.1)	71.6 (10.4)	11.102	<0.001
Alanine aminotransferase (U/L)	19.0 (14.0–27.0)	14.0 (11.0–19.0)	14.849^b^	<0.001
*γ*-Glutamyltransferase	23.0 (15.0–39.0)	15.0 (11.0–22.0)	16.215^b^	<0.001
Triglyceride (mmol/L)	1.32 (0.89–1.94)	0.89 (0.67–1.24)	16.255^b^	<0.001
Total cholesterol (mmol/L)	4.77 (4.15–5.50)	4.51 (3.98–5.08)	7.189^b^	<0.001
HDL cholesterol (mmol/L)	1.27 (0.34)	1.40 (0.32)	10.076	<0.001
LDL cholesterol (mmol/L)	2.67 (0.73)	2.50 (0.64)	6.479	<0.001
Fasting plasma glucose (mmol/L)	4.83 (4.53–5.23)	4.69 (4.44–4.95)	8.945^b^	<0.001
Serum uric acid (*μ*mol/L)	327.6 (85.0)	284.7 (74.4)	13.986	<0.001
Serum sialic acid (mg/dL)	61.1 (7.3)	58.2 (6.9)	10.263	<0.001

Data are expressed as mean (SD) or median (IQR).

^a^ *χ* ^2^ value; ^b^ *Z* value; HDL: high-density lipoprotein; LDL: low-density lipoprotein.

**Table 2:** Association of serum sialic acid with prevalence rate of NAFLD.

Sialic acid quartiles	Total	NAFLD	PR%	PR	*χ* ^2^	*P*
Quartile 1	1459	106	7.27	1.00		
Quartile 2	1474	132	8.96	1.23		
Quartile 3	1502	178	11.85	1.63		
Quartile 4	1481	277	18.70	2.57	108.758	<0.001

PR%: prevalence rate; PR: prevalence ratio.

**Table 3:** Correlations between serum sialic acid and features of metabolic syndrome.

	WC	SBP	DBP	TG	HDL-C	FBG
*r* value	0.196	0.175	0.168	0.212	−0.077	0.159
*P* value	<0.001	<0.001	<0.001	<0.001	<0.001	<0.001

DBP: diastolic blood pressure; FBG: fasting blood glucose; HDL-C: high-density lipoprotein cholesterol; SBP: systolic blood pressure; TG: triglyceride; WC: waist circumference.

**Table 4:** Risk factors associated with the presence of NAFLD.

Variables	*β*	SE	Wald *χ* ^2^	*P*	OR (95% CI)
Age (years)	0.018	0.004	19.279	<0.001	1.018 (1.010–1.026)
Body mass index (kg/m^2^)	0.137	0.040	11.628	0.001	1.147 (1.060–1.240)
Waist circumference (cm)	0.063	0.009	52.989	<0.001	1.065 (1.047–1.083)
Alanine aminotransferase (U/L)	0.004	0.001	10.426	0.001	1.004 (1.002–1.007)
Triglyceride (mmol/L)	0.195	0.041	22.508	<0.001	1.216 (1.122–1.318)
HDL cholesterol (mmol/L)	−0.577	0.154	14.080	<0.001	0.562 (0.416–0.759)
Fasting plasma glucose (mmol/L)	0.218	0.038	32.222	<0.001	1.243 (1.153–1.341)
Serum uric acid (*μ*mol/L)	0.003	0.001	24.556	<0.001	1.003 (1.002–1.004)
Serum sialic acid (mg/dL)	0.018	0.006	9.369	0.002	1.018 (1.007–1.030)

*β*: partial regression coefficient; SE: standard error of partial regression coefficient; OR: odds ratio; CI: confidence interval; HDL: high-density lipoprotein.
